# Xanthine oxidase inhibitory study of eight structurally diverse phenolic compounds

**DOI:** 10.3389/fnut.2022.966557

**Published:** 2022-09-20

**Authors:** Arshad Mehmood, Jiayi Li, Ashfaq Ur Rehman, Rovina Kobun, Inam U Llah, Imran Khan, Fayez Althobaiti, Sarah Albogami, Muhammad Usman, Fahad Alharthi, Mohamed Mohamed Soliman, Sanabil Yaqoob, Kanza Aziz Awan, Liang Zhao, Lei Zhao

**Affiliations:** ^1^Beijing Advanced Innovation Center for Food Nutrition and Human Health, Beijing Engineering and Technology Research Center of Food Additives, School of Food and Health, Beijing Technology and Business University, Beijing, China; ^2^Department of Food Science and Technology, University of Haripur, Haripur, Pakistan; ^3^State Key Laboratory of Microbial Metabolism, Joint International Research Laboratory of Metabolic and Developmental Sciences, School of Life Sciences and Biotechnology, Shanghai Jiao Tong University, Shanghai, China; ^4^Faculty of Food Science and Nutrition, Universiti Malaysia Sabah, Kota Kinabalu, Malaysia; ^5^Department of Biotechnology, College of Science, Taif University, Taif, Saudi Arabia; ^6^Department of Biology, College of Science, Taif University, Taif, Saudi Arabia; ^7^Clinical Laboratory Sciences Department, Turabah University College, Taif University, Taif, Saudi Arabia; ^8^Department of Food Science and Technology, Faculty of Science and Technology, University of Central Punjab, Lahore, Pakistan

**Keywords:** xanthine oxidase, gout, polyphenols, uric acid, atomic force microscopy

## Abstract

This project was designed to explore the xanthine oxidase (XO) inhibitory mechanism of eight structurally diverse phenolic compounds [quercetin: C1, quercetin-3-rhamnoside: C2, 4, 5-O-dicaffeoylquinic acid: C3, 3, 5-O-dicaffeoylquinic acid: C4, 3, 4-O-di-caffeoylquinic acid: C5, 4-O-caffeoylquinic acid (C6), 3-O-caffeoylquinic acid: C7, and caffeic acid: C8]. For this purpose, *in-vitro* and different computational methods were applied to determine the xanthine oxidase (XO) inhibitory potential of eight structurally diverse phenolic compounds. The results revealed that phenolic compounds (C1–C8) possess strong to weak XO inhibitory activity. These results were further confirmed by atomic force microscopy (AFM) and ^1^H NMR analysis. Furthermore, computational study results revealed that phenolic compounds (C1–C8) bind with the surrounding amino acids of XO at the molybdenum (MO) site. These *in-vitro* and *in-silico* results divulge that phenolic compounds have a strong potential to lower uric acid levels *via* interacting with the XO enzyme and can be used to combat hyperuricemia.

## Introduction

Xanthine oxidase (XO, EC 1.17.3.2) enzyme abundantly present in two interconvertible forms xanthine oxidoreductase (XOR), and xanthine dehydrogenase (XDH) in milk, animal, and human cells contains two molecules of FAD (flavin adenine dinucleotide), 1,330 amino acids, eight iron atoms, and two molybdenum atoms. It is an important enzyme for the catalysis of xanthine to hypoxanthine and hypoxanthine to uric acid (UA). The molybdenum cofactor is the active site of this enzyme, whereas the iron atom (2Fe-2S) helps for the electron transfer system (1–4). The overactivity of XO is directly linked with excessive UA production. The higher UA production or under excretion results in hyperuricemia (HUA), gout, and many other diseases (5).

The lifestyle, genetic makeup, age, and dietary habits (consumption of a more purine-rich diet e.g., meat, seafood, and sugar) are directly associated with the increment of UA level. The prevalence rate of HUA significantly elevated worldwide due to changes in lifestyle (6). To overcome the burden of body UA level, inhibiting key enzyme XO is an effective clinical approach. Allopurinol and febuxostat have been used for the treatment of gout and HUA (6–8). Notably, all these drugs persist with side effects, such as fever, abdominal pain, skin rashes, allergic diarrhea, and others that can damage the liver (9, 10). Hence, an alternative XO inhibitor with fewer side effects for treating HUA-related disorders is in dire need.

Natural products have been considered as an important source of bioactive compounds. It has been reported that a diet rich with bioactive compounds exerts a protective effect against many diseases, including HUA. Polyphenols, especially chlorogenic acid compounds, are naturally occurring compounds abundantly present in edible food plants, such as fruits, vegetables, tea, coffee, and cereals (11, 12). For example, whole apples have been reported to contain 62–358 mg of chlorogenic acid compounds per kg of fresh matter. Coffee beans are the richest dietary source of chlorogenic acid compounds, with a total chlorogenic acid compound content of 6–12% for the dry weight of coffee beans (13). The polyphenolic compounds have been reported to overcome the burden of HUA *via* blocking the XO enzyme (14–16).

This study was designed to explore the inhibition mechanism of eight structurally diverse phenolic compounds commonly present in fruit plants (quercetin, quercetin-3-rhamnoside, 4,5-O-dicaffeoylquinic acid, 3,5-O-dicaffeoylquinic acid, 3,4-O-di-caffeoylquinic acid, 4-O-caffeoylquinic acid, 3-O-caffeoylquinic acid, and caffeic acid) on XO by using ^1^H NMR, atomic force microscopy (AFM), and various computational techniques. This study will pave the way for the development of safe functional components from food sources for the treatment of UA-related disorders, such as HUA and gout.

## Results

### *In vitro* xanthine oxidase inhibitory and structure-activity relationship of phenolic compounds with XO

In this study, eight polyphenolic compounds including two flavonoids and six chlorogenic acids were screened against XO inhibitory activity. The polyphenolic compounds showed a concentration-dependent inhibition. The IC_50_ values of tested compounds (C1–C8) are presented in Table 1. As shown in Table 1, flavonoid compounds (C1 and C2) possessed more potent XO inhibitory activity compared to chlorogenic acids (C3–C8) compounds. Notably, phenolic compounds (C1–C8) exhibited lower inhibitory activity.

**Table 1:** Xanthine oxidase (XO) inhibitory activity of phenolic compounds.

**Compounds**	**IC_50_ (μM)**	**Inhibition type**
C1 (Quercetin)	6.45 ± 0.26^hi^	Mixed
C2 (Quercitrin)	12.09 ± 0.65^g^	Competitive
C3 (4,5-Di-O-caffeoylquinic acid)	26.74 ± 1.20^ef^	Mixed
C4 (3,5-Di-O-caffeoylquinic acid)	28.90 ± 1.02^e^	Competitive
C5 (3,4-O-Dicaffeoylquinic acid)	44.57 ± 1.65^d^	Competitive
C6 (4-O-Caffeoylquinic acid)	70.23 ± 2.25^c^	Competitive
C7 (Chlorogenic acid)	80.93 ± 3.06^b^	Competitive
C8 (Caffeic acid)	95.65 ± 3.28^a^	Competitive

The IC_50_ values are presented as the mean ± SD, and different letters represent the difference (p < 0.05) by one-way ANOVA.

The chlorogenic acid compounds (C3–C8) are all esters of caffeic acid and quinic acid, and in their structure quinic acid was a core molecule and one to two caffeic acids conjugated with the quinic acid. Previously, study results showed that caffeic acid and quinic acid exhibited week XO inhibitory activity. In this study, we also noticed week XO inhibitory activity of caffeic acid (C8). It can be speculated that XO inhibitory activity of chlorogenic acid compounds does not rely on the quinic or caffeoyl group. Their conjunction side might play an important role in affecting XO activity. Notably, three di chlorogenic acids (C3, C4, and C5) showed more profound XO inhibitory activity concerning their mono chlorogenic acids (C6 and C7) and caffeic acid (C8). The caffeoyl group consists of two hydroxyl groups and a conjugated ring, which are closely linked with XO inhibition. The compounds C3 and C4 exhibited higher XO inhibitory than C5 due to their binding difference of caffeoyl groups on the quinic core. The caffeoyl moiety at position 3 decreases XO inhibitory activity, whereas at position 5 enhances inhibitory activity.

### ^1^H NMR titration study

**Figure 1:**
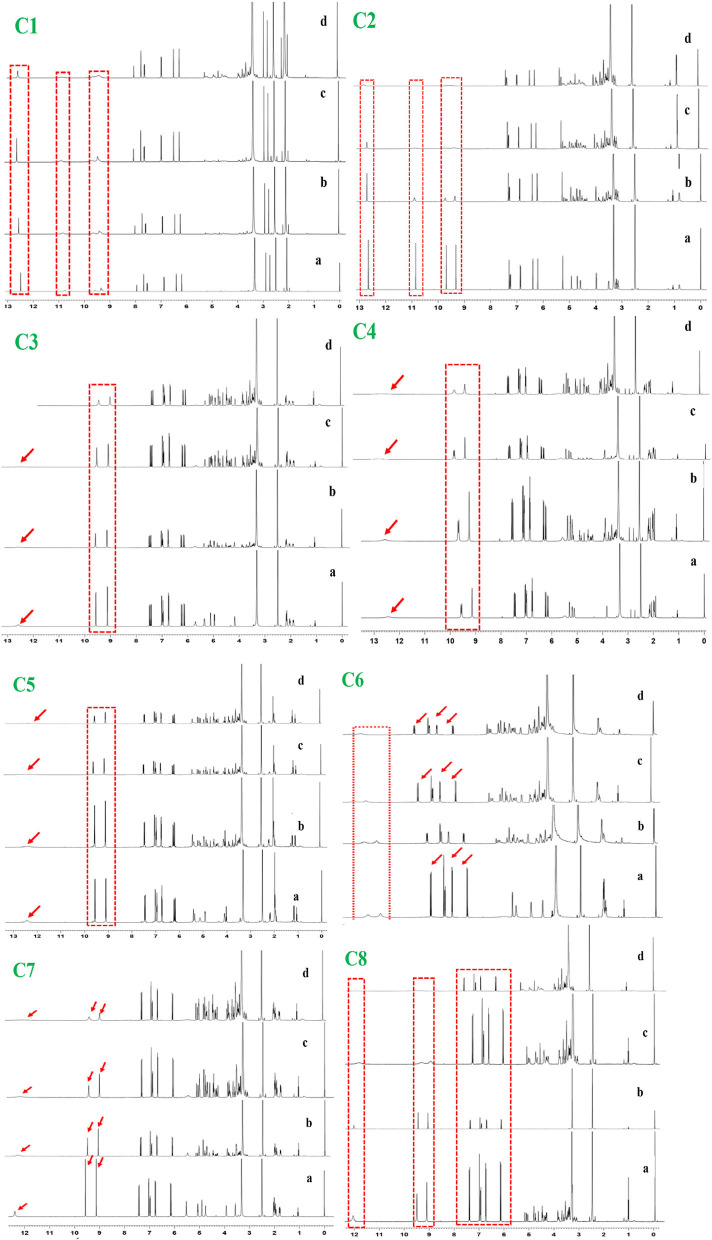
^1^H NMR analysis of phenolic compounds with XOD. C1, quercetin; C2, quercetin-3-rhamnoside; C3, 4,5-O-dicaffeoylquinic acid; C4, 3,5-O-dicaffeoylquinic acid; C5, 3,4-O-di-caffeoylquinic acid; C6, 4-O-caffeoylquinic acid; C7, 3-O-caffeoylquinic acid; C8, caffeic acid. The letter a**–**d from each figure means the various concentrations of XO (0.1**–**0.5 mg/μl). ^1^H NMR: Proton nuclear magnetic resonance.

^1^H NMR titration is an important method to study the interaction of various molecules by observing the change in the chemical shift of hydrogen atoms. The interaction of phenolic compounds (C1–C8) with XO was further determined by using a ^1^H NMR titration assay in DMSO-d6 solvent. The ^1^H NMR titration was performed by the addition of different concentrations of XO to inhibitor compounds and their corresponding ^1^H NMR plots were constructed (Figure 1). The flavonoid compounds (C1 and C2) significantly showed changes in their chemical shifts at ring A [C1, 7OH (9.68–9.39) and 5OH (10.94–10.65 ppm)], [C2, 7OH (10.86) and 5OH (12.66 ppm)], ring B [C1, 3OH (9.39 ppm) and 4OH (9.20 ppm), C2, 3OH (9.33 ppm) and 4OH (9.7 ppm)], and ring C [C1, 3OH (9.39 ppm) and C2, 4OH (9.70 ppm)]. However, additional benzene ring (methylation and OH) in compound C2 showed nonsignificant chemical shift changes. Similarly, chlorogenic acid compounds (C3–C8) also showed profound chemical shift changes in their structure at caffeoyl (3, 4 OH) and quinic acid (3, 4 OH) core. Interestingly, reduction in NMR peaks at COOH in quinic acid structure was also observed.

### AFM analysis

**Figure 2:**
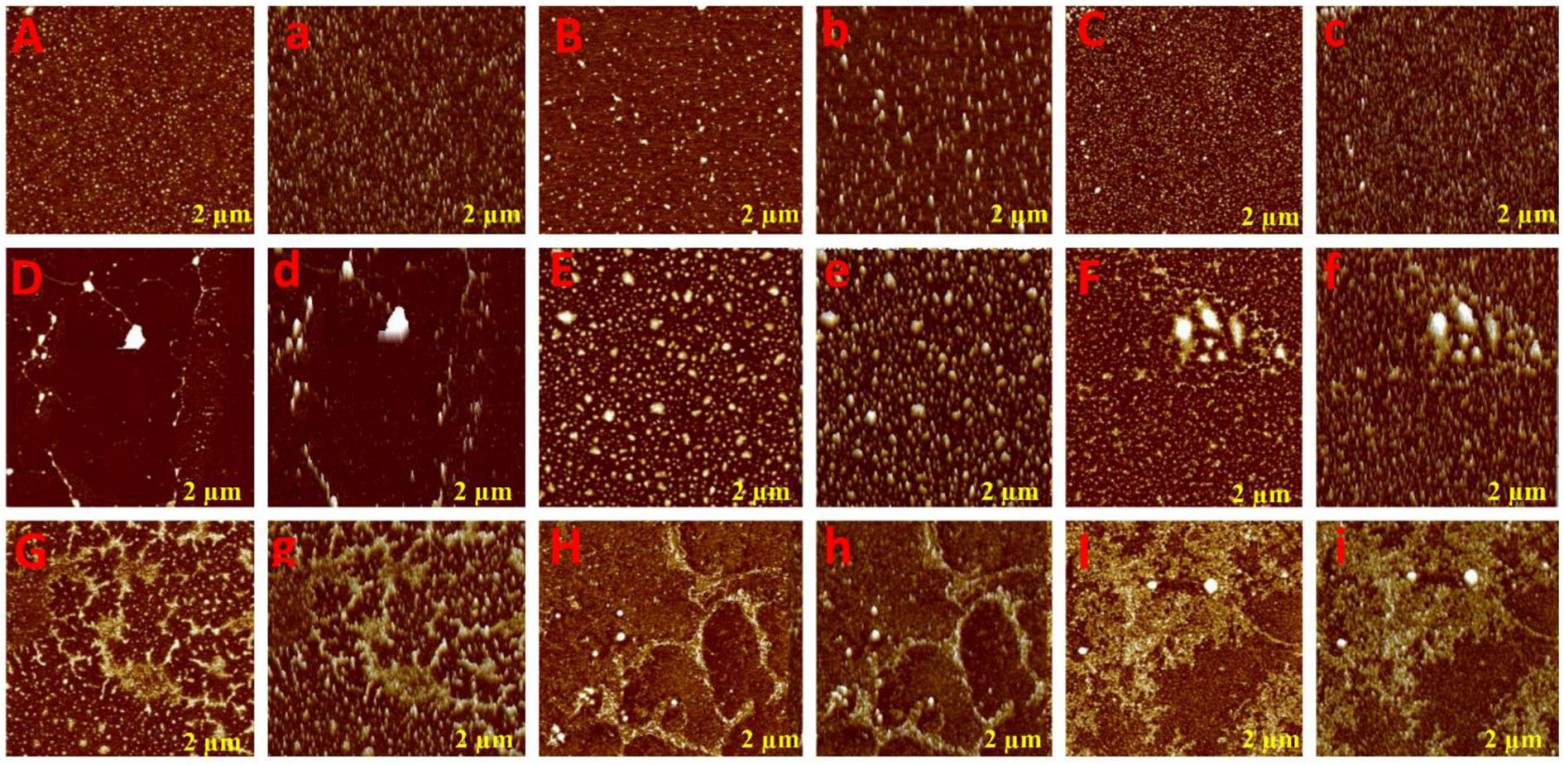
Atomic force microscopic analysis of phenolic compounds with XOD. Aa (XOD only), Bb (C8: 3-O-caffeoylquinic acid), Cc (C7: 4-O-caffeoylquinic acid), Dd (C6: caffeic acid), Ee (C5: 3,4-O-dicaffeoylquinic acid), Ff (C4: 3,5-O-dicaffeoylquinic acid), Gg (C3: 4,5-O-dicaffeoylquinic acid), Hh (C2: quercetin-3-O-rhamnoside), and Ii (C1: quercetin).

To glean further interaction of phenolic compounds (C1–C8) with XO, we applied the AFM technique in the Tris-HCl buffer system (pH 7.40), which is widely used for biomolecular structural studies. As shown in the topography image (Figure 2), the white spot represents free XO that was uniformly distributed on the mica surface. However, after the addition of phenolic compounds (C1–C8), the XO structure was disturbed and formed a new stable structure (fibril network). As seen in Figure 2, all compounds formed different fibril networks which indicated their interaction was not identical due to structural differences. However, compounds C1–C4 formed a more stable structure (fibril network) that consists of their inhibitory activity. Generally, free XO may be uniformly distributed in the solution by interacting with various molecular forces (hydrogen bond, hydrophobic interaction, etc). However, the addition of phenolic compounds (C1–C8) to XO solution, results in changes in the surrounding environment of XO which further leads to XO molecules being exposed to a more hydrophobic environment. Hence, the XO molecule on mica becomes large and bigger, which represents that hydrophobic interaction occurred among phenolic compounds (C1–C8) and XO.

### Docking results

**Figure 3:**
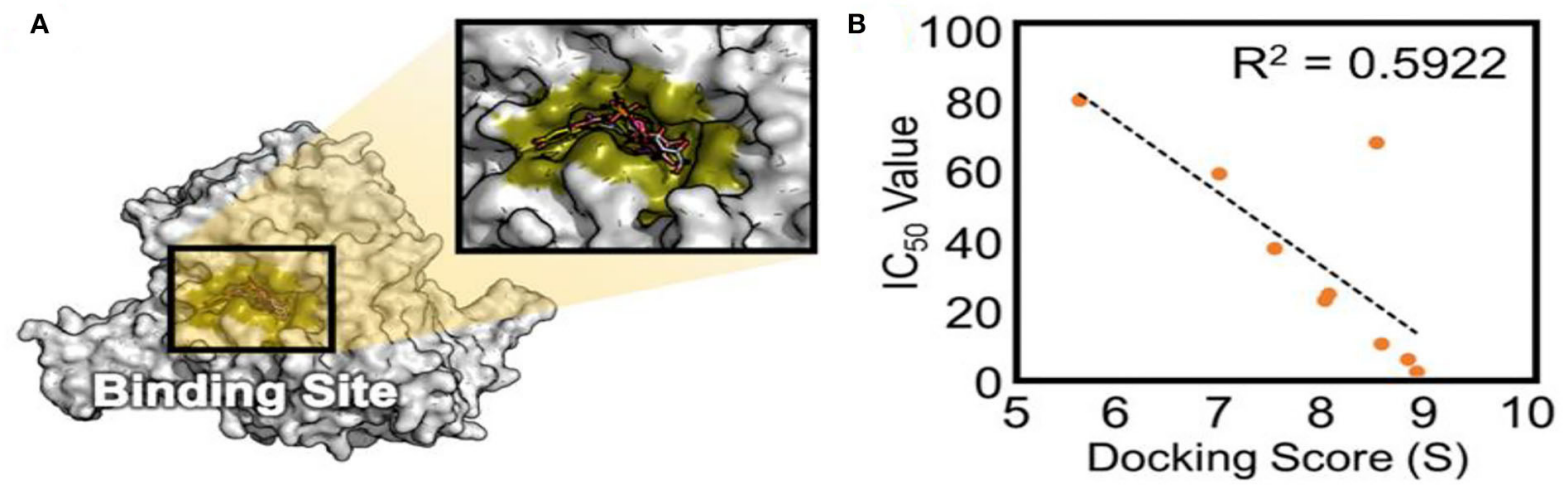
The active site of XO **(A)** and experimentally determined IC_50_ values **(B)** of different phenolic compounds shown concerning their ranking by docking score. XO, Xanthine oxidase.

**Figure 4:**
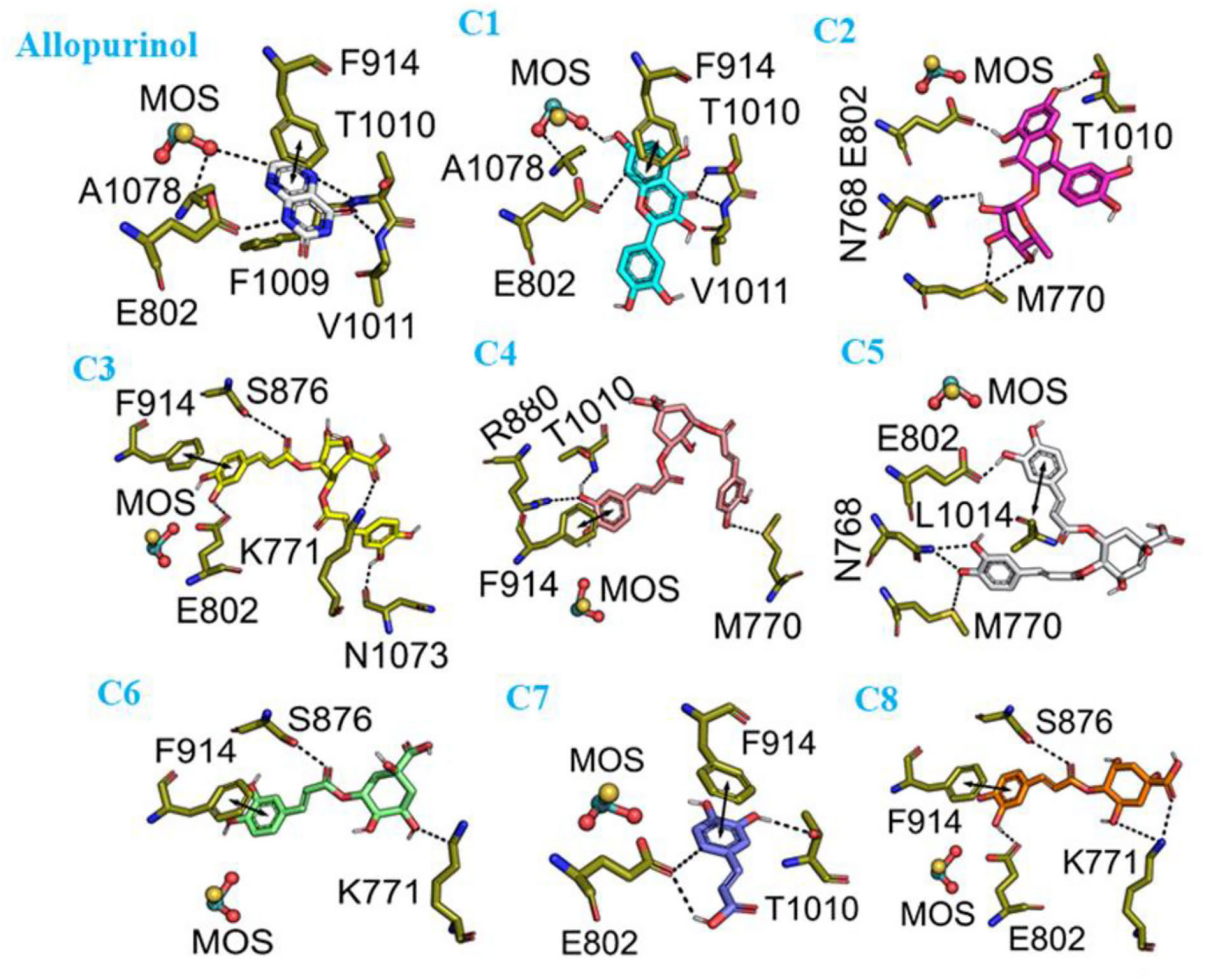
The molecular docking interaction of phenolic compounds (C1–C8) with xanthine oxidase (XO). C1, quercetin; C2, quercetin-3-rhamnoside; C3, 4,5-O-dicaffeoylquinic acid; C4, 3,5-O-dicaffeoylquinic acid; C5, 3,4-O-di-caffeoylquinic acid; C6, 4-O-caffeoylquinic acid; C7, 3-O-caffeoylquinic acid; C8, caffeic acid.

To validate the docking method and docking accuracy, the chemical structures, biological activity values, and docking scores are shown in Table 2. Several of these compounds had IC_50_ values in the range of (6.25–95.65 μM) as determined via an interaction assay. The correlation between docking scores and biological activity (IC_50_ values) is presented in Figures 3, 4. They are quite correlated quantitatively with their experimentally determined activity. The above results indicated acceptable reliability of the docking method for the XO receptor and phenolic compounds. The phenolic compound is tightly bound with the surrounding amino acids, such as the C1 (F914, T1010, V1011, E802, and A1078), C2 (E802, N768, M770, and T1010), C3 (F914, S876, K771, N1073, and F914), C4 (T1010, M770, F914, and R880), C5 (E802, L1014, N68, and M770), C6 (F914, S876, and K771), C7 (914, T101, and E802), and C8 (F914, S876, and K771).

**Table 2:** Interaction detail of phenolic compounds with xanthine oxidase enzyme.

**Phenolic compounds**	**Ligand**	**Receptor**	**Interaction**	**Distance**	**E (kcal/mol)**
C1	O5	S MOS1327	H-donor	4.02	−1.4
	C15	OE2 GLU802	H-donor	3.51	−0.5
	O4	N THR1010	H-acceptor	3.03	−0.8
	O4	OG1 THR1010	H-acceptor	2.76	−1.5
	O4	N VAL1011	H-acceptor	3.11	−1.2
	6-ring	6-ring PHE914	π-π	3.41	−0.0
C2	O3	SD MET770	H-donor	3.05	−1.8
	O4	SD MET770	H-donor	3.51	−0.6
	O8	OE1 GLU802	H-donor	2.52	−4.2
	O9	OG1 THR1010	H-donor	3.03	−0.7
	O5	ND2 ASN768	H-acceptor	2.89	−1.3
C3	O9	O ASN1073	H-donor	2.81	−1.7
	O10	OE1 GLU802	H-donor	2.93	−4.7
	O10	OE2 GLU802	H-donor	3.09	−0.6
	O6	NZ LYS771	H-acceptor	3.14	−4.3
	O8	OG SER876	H-acceptor	3.55	−0.5
	6-ring	6-ring PHE914	π-π	3.87	−0.0
C4	O9	OG1 THR1010	H-donor	3.38	−0.9
	O12	SD MET770	H-donor	4.03	−1.5
	O9	NH2 ARG880	H-acceptor	3.46	−0.7
	6-ring	6-ring PHE914	π-π	3.80	−0.0
C5	O9	OE1 GLU802	H-donor	2.68	−5.7
	O12	SD MET770	H-donor	4.41	−1.2
	O10	ND2 ASN768	H-acceptor	2.96	−0.8
	O12	ND2 ASN768	H-acceptor	3.07	−0.5
	6-ring	CD2 LEU1014	pi-H	3.57	−0.5
C6	O8	OE1 GLU802	H-donor	2.94	−4.6
	O8	OE2 GLU802	H-donor	3.06	−0.7
	O3	CE LYS771	H-acceptor	3.73	−0.6
	O6	NZ LYS771	H-acceptor	3.03	−2.8
	O7	OG SER876	H-acceptor	3.33	−1.2
	6-ring	6-ring PHE914	π-π	3.84	−0.0
C7	O4	CE LYS771	H-acceptor	3.15	−1.3
	O7	OG SER876	H-acceptor	2.69	−2.2
	6-ring	6-ring PHE914	π-π	3.41	−0.0
C8	O1	OG1 THR1010	H-donor	3.54	−0.5
	O3	OE1 GLU802	H-donor	3.34	−0.6
	C7	OE1 GLU802	H-donor	3.38	−0.5
	6-ring	6-ring PHE914	π-π	3.43	−0.0
Allopurinol	N1 1	OE1 GLU802	H-donor	2.94	−10.4
	C9A 10	O1 MOS1327	H-donor	3.85	−0.6
	O4 7	N VAL1011	H-acceptor	3.05	−1.2
	N5 9	N THR1010	H-acceptor	3.57	−0.7

C1, quercetin; C2, quercetin-3-rhamnoside; C3, 4,5-O-dicaffeoylquinic acid; C4, 3,5-O-dicaffeoylquinic acid; C5, 3,4-O-di-caffeoylquinic acid; C6, 4-O-caffeoylquinic acid; C7, 3-O-caffeoylquinic acid; and C8, caffeic acid.

### Evaluation of the stability of allopurinol and phenolic compounds with XO

**Figure 5:**
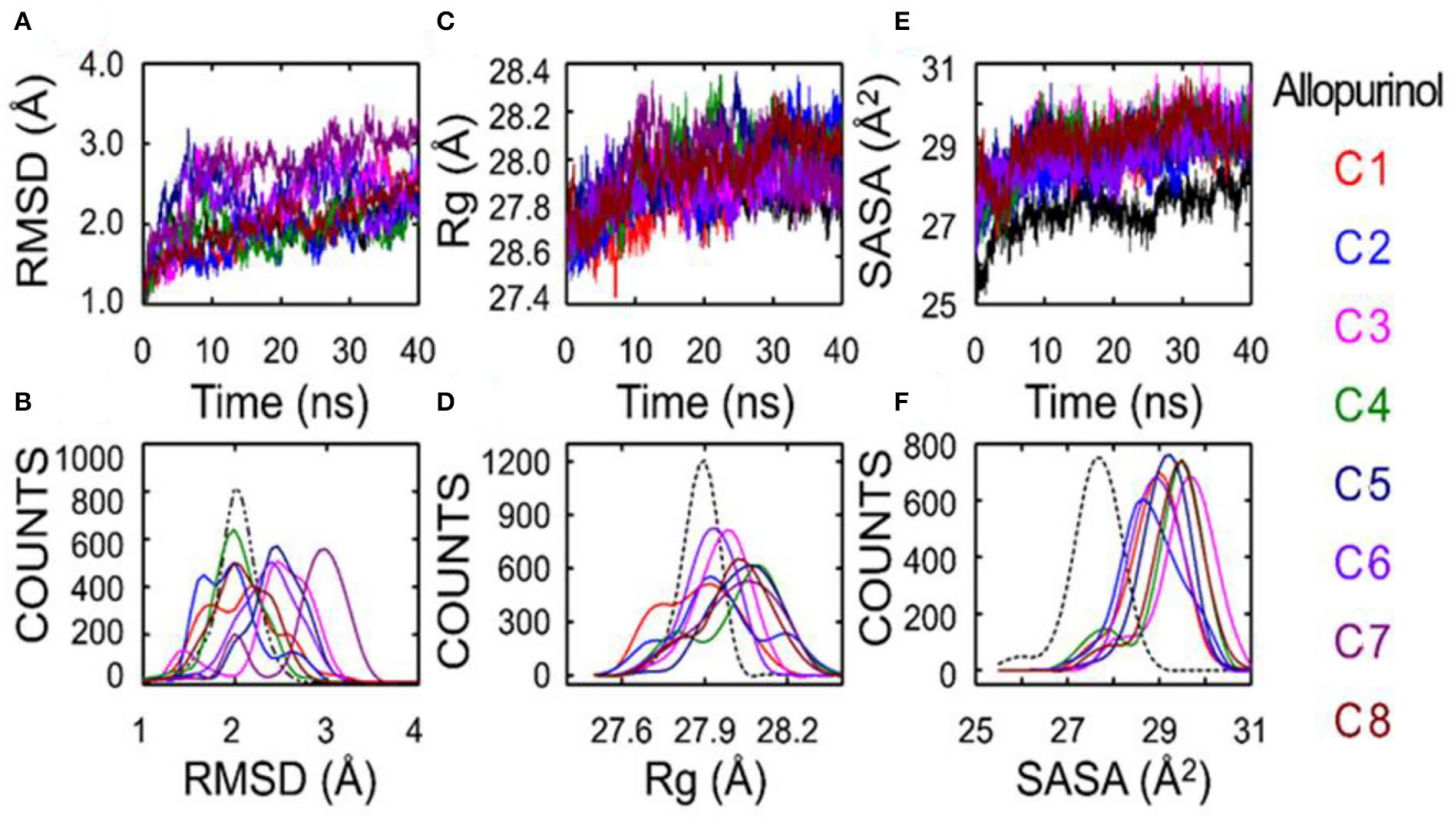
Superposed plots for the RMSD, radius of gyration (Rg), and SASA of allopurinol–XO and phenolic compounds–XO. **(A)** Superposed RMSD graph, **(B)** The frequency distribution graph (RMSD vs. frequency counts), **(C)** Superposed Rg graph, **(D)** The frequency distribution graph (Rg vs. frequency counts), **(E)** Schematic representation of the solvent-accessible surface area (SASA), and **(F)** Distribution of SASA. SASA, solvent accessible surface area; RMSD, root-mean-square deviation; C1, quercetin; C2, quercetin-3-rhamnoside; C3, 4,5-O-dicaffeoylquinic acid; C4, 3,5-O-dicaffeoylquinic acid; C5, 3,4-O-di-caffeoylquinic acid; C6, 4-O-caffeoylquinic acid; C7, 3-O-caffeoylquinic acid; and C8, caffeic acid.

The results regarding root-mean-square deviation (RMSD) analysis showed that the average backbone of allopurinol and eight phenolic compounds (C1–C8) were found to differ between 1.5 and 3.0Å and then became stable during the whole molecular dynamics (MD) simulation period (Figure 5). The C1–C8 complexes exhibited a slight increment and further decrease in comparison to a positive control (allopurinol). The stable RMSD of XO till the end of simulation predicted that MD simulation is right and can be further used for rigorous analysis. The higher RMSD fluctuation during the simulation was observed in the compound 7 complex. After that, compound 7 complex was stable at ~3Å average backbone RMSD. The high swing of some compound complexes, such as C7 and C8, in the backbone of RMSD disclose that these compounds are unstable may be due to weak interactions with surrounding residue. Additionally, the compactness of ligand–protein was determined by using a radius of gyration (R_g_) method. If a protein is stably folded, it will likely maintain a relatively steady value of R_g_, whereas it will change over time for unfolded proteins (17). The results showed that Rg of allopurinol and C1–C8 compound complexes was not the same. From the computed line graphs of distance distribution, we noticed that the results of Rg of allopurinol and C1–C8 complexes exhibited a slight difference in their steadiness (Figures 5C,D); however, similar Rg values (around 28Å^2^) during MD simulation time indicated the stable conformation. The allopurinol had a lower value of Rg, which predicted that more strongly interactions with surrounding residues of XO lead to stronger structure stability with comparisons to the C1–C8 complexes (high Rg value).

**Figure 6:**
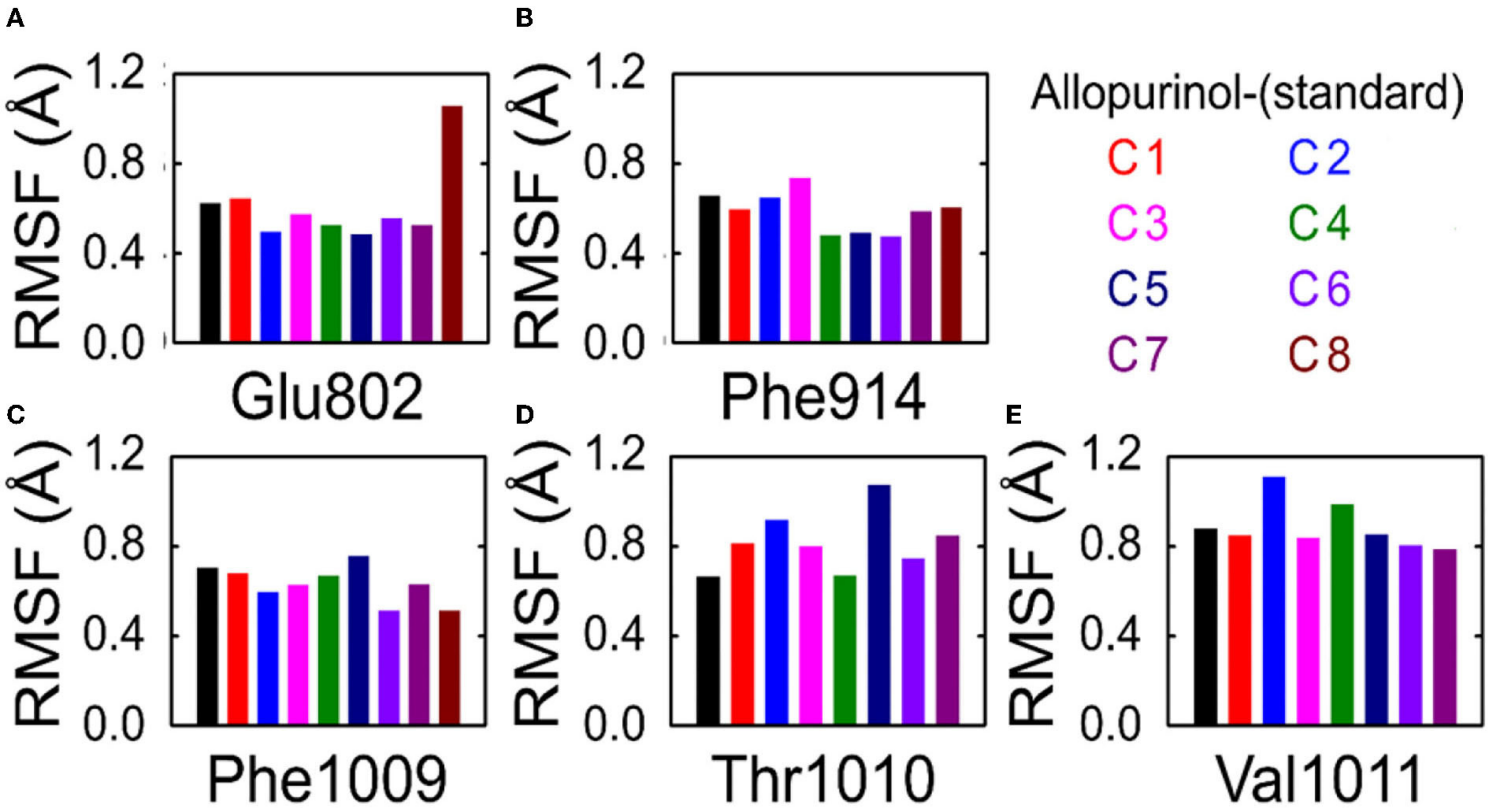
RMSF analysis of key residues in allopurinol and phenolic compounds. **(A)** Glu802; **(B)** Phe914; **(C)** Phe1009; **(D)** Thr1010; and **(E)** Val1011. RMSF, root-mean-square fluctuations; C1, quercetin; C2, quercetin-3-rhamnoside; C3, 4,5-O-dicaffeoylquinic acid; C4, 3,5-O-dicaffeoylquinic acid; C5, 3,4-O-di-caffeoylquinic acid; C6, 4-O-caffeoylquinic acid; C7, 3-O-caffeoylquinic acid; and C8, caffeic acid.

To understand the effect of individual amino acids binding with allopurinol and bound with other compounds (C1–C8), we analyzed the Cα root-mean-square fluctuations (RMSF) of these key residues. There was slight residue fluctuation in regions adjacent to the mutation site. For residue GLU802, except for complex 8 which showed a particularly high RMSF, the RMSF values of the other compounds were similar, about 0.6Å (Figure 6).

### Effect of phenolic compounds on solvent accessible surface accessibility at the catalytic site of XO

Theoretically, changes in the accessibility of protein to solvent can be determined by computing solvent accessible surface area (SASA). The SASA parameter can be used for determining the accessibility of protein to solvent (18). Consequently, the computed line graphs of SASA distribution revealed that the value of allopurinol was the smallest, <28Å^2^ (Figures 5E,F). On the contrary, the other eight different systems exhibited different traits the enzyme and compounds moderately moved away from each other, and this movement further facilitate the increment of SASA. The SASA values of the C1–C8 systems exceeded 28Å^2^, which were basically in the range of 28–30Å^2^. The SASA values suggested that during the weakening of contacts of interface residues the SASA interface region will increase significantly. The outcome indicated that the “open state” leads to a decrease in binding affinity. The conformation shifts from the “closed” state (allopurinol) toward the “open” state as the simulation progresses. The gradual transition from the “open” to the “closed” states could imply the disappearance of the interaction (Table 2).

### Noncovalent interaction between key residues and phenolic compounds (C1–C8)

**Figure 7:**
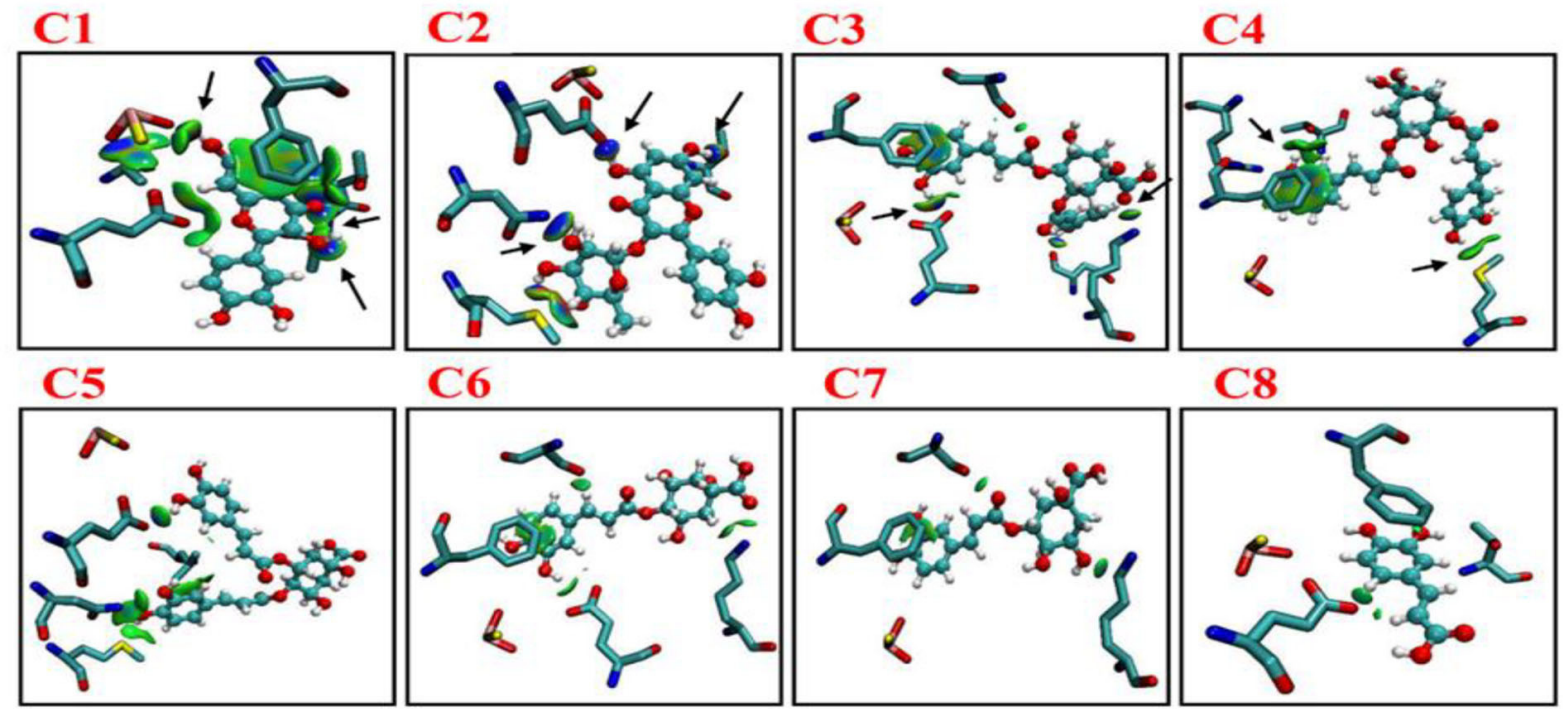
Intermolecular interactions (isosurfaces: 0.01 a.u.) for different models using IGM analysis (blue represents a strong attraction, and green denotes a weak repulsion). C1, quercetin; C2, quercetin-3-rhamnoside; C3, 4,5-O-dicaffeoylquinic acid; C4, 3,5-O-dicaffeoylquinic acid; C5, 3,4-O-di-caffeoylquinic acid; C6, 4-O-caffeoylquinic acid; C7, 3-O-caffeoylquinic acid; and C8, caffeic acid.

According to distance analysis in the allopurinol–XO system, the interface residues held the atomic interaction (Van der wall interaction and hydrogen bonds). While the point interaction, some residues lost the strong interactions (e.g., E802 and T1010). Furthermore, the results of non-covalent interaction correlate with the analysis of the essential dynamics. The independent gradient model (IGM) was used for the dominant structure of different complexes. One of the advantages of the IGM method over the popular NCI method is that: intra and interfragment linkage might be individually studied. The green oval represents the favorable interaction between the fragments and can be regarded as van der Waals interaction, whereas stronger stabilizing/destabilizing interactions are represented by blue/red color. From Figure 7, it can be seen that there was strong interaction among the compounds (C1–C8) and surroundings residues. In Figure 7, the area of the linkage region might be used as an indication for the extent of bonding. In C1–C3, there was a large interaction region between C1 and C3 and residues nearby. For the C1 system, it was observed that a vast green oval region between C1 and MOS, F914, E802, T1010, and V1011. Especially, O1_MOS_, N_T1010_, OG1_T1010_, and CB_V1011_ formed the hydrogen bond with C1 since the blue ovals appeared.

In the C2 system, ND2_N768_, OE1_E802_, and OG1_T1010_ formed hydrogen bonds with C2. In the C3 system, OE1_E802_ and NZ_K771_ formed a hydrogen bond with C3. As shown in Figure 7, strong linkage/interactions are exhibited as blue isosurface area. On the contrary, C4–C8 compounds exhibited different behavior and moderately moved away from adjacent residues, and thus, this slight movement disrupted the interaction, showing less area of a green oval. The compounds (C4–C8) showed interactions of only weak noncovalent forces e.g., London (dispersion) and van der Waals forces (Figure 7).

### Hirshfeld surface analysis

**Figure 8:**
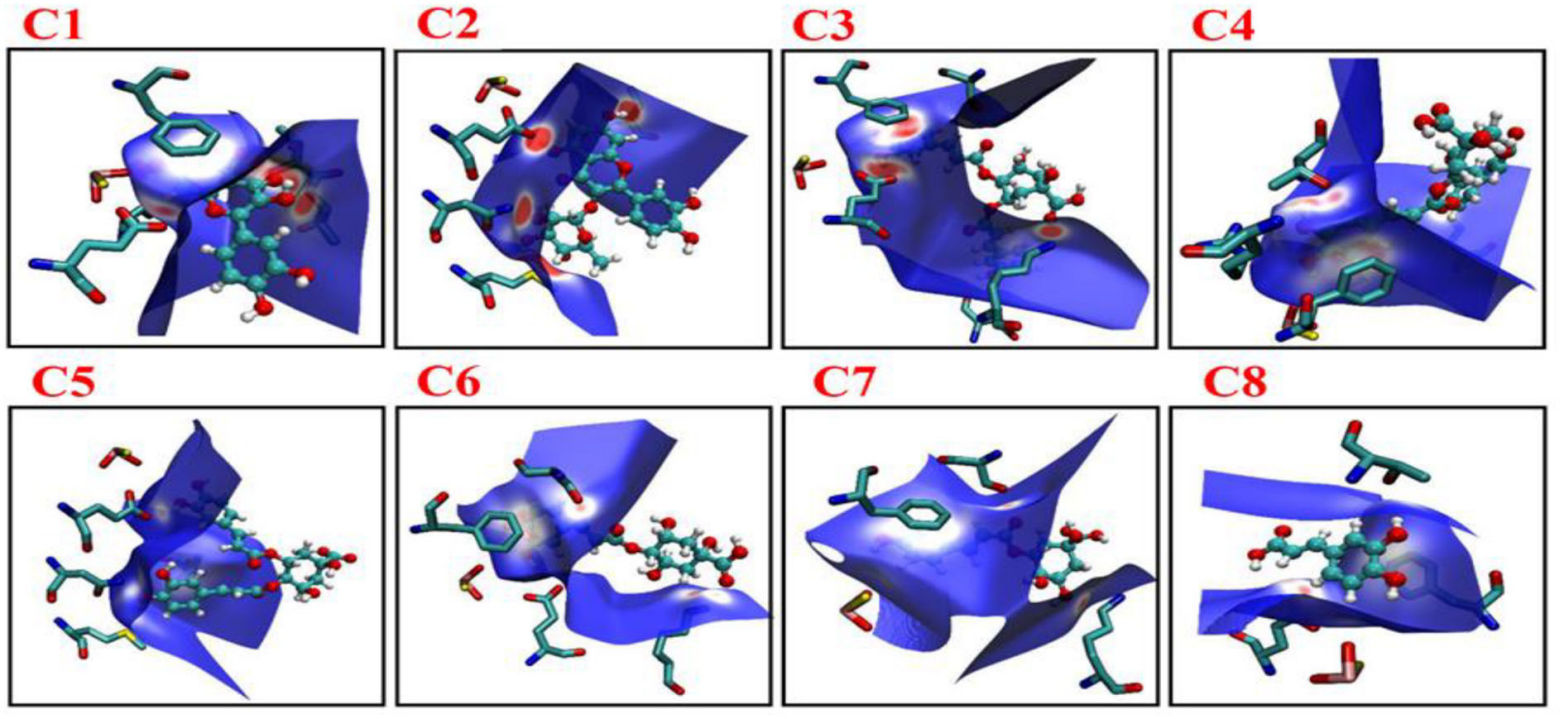
Images showing the Hirshfeld surfaces that use color-coding to represent the proximity of close contacts around residues in phenolic compounds C1–C8 in different system (white, distance *d* equals the van der Waals distance; blue, *d* exceeds the van der Waals distance; red, *d* is less than van der Waals distance). C1, quercetin; C2, quercetin-3-rhamnoside; C3, 4,5-O-dicaffeoylquinic acid; C4, 3,5-O-dicaffeoylquinic acid; C5, 3,4-O-di-caffeoylquinic acid; C6, 4-O-caffeoylquinic acid; C7, 3-O-caffeoylquinic acid; and C8, caffeic acid.

The non-covalent interaction was also determined by Hirshfeld surface analysis. The Hirshfeld surface analysis gives a deeper insight into the nature of intermolecular interactions. In Figure 8, MOS, F914, E802, T1010, and V1011 formed stronger interactions with C1 (red area short distance) from Hirshfeld surface. The effect of these interactions in altering the structure-directing pairwise interactions observed in C1, C2, and C3 is of particular interest and has implications for the appearance of hydrogen-bonded structures. The strong and weaker interaction was represented by blue and white regions on the surface (C4–C8) in Figure 8.

### Reduced density gradient analysis

**Figure 9:**
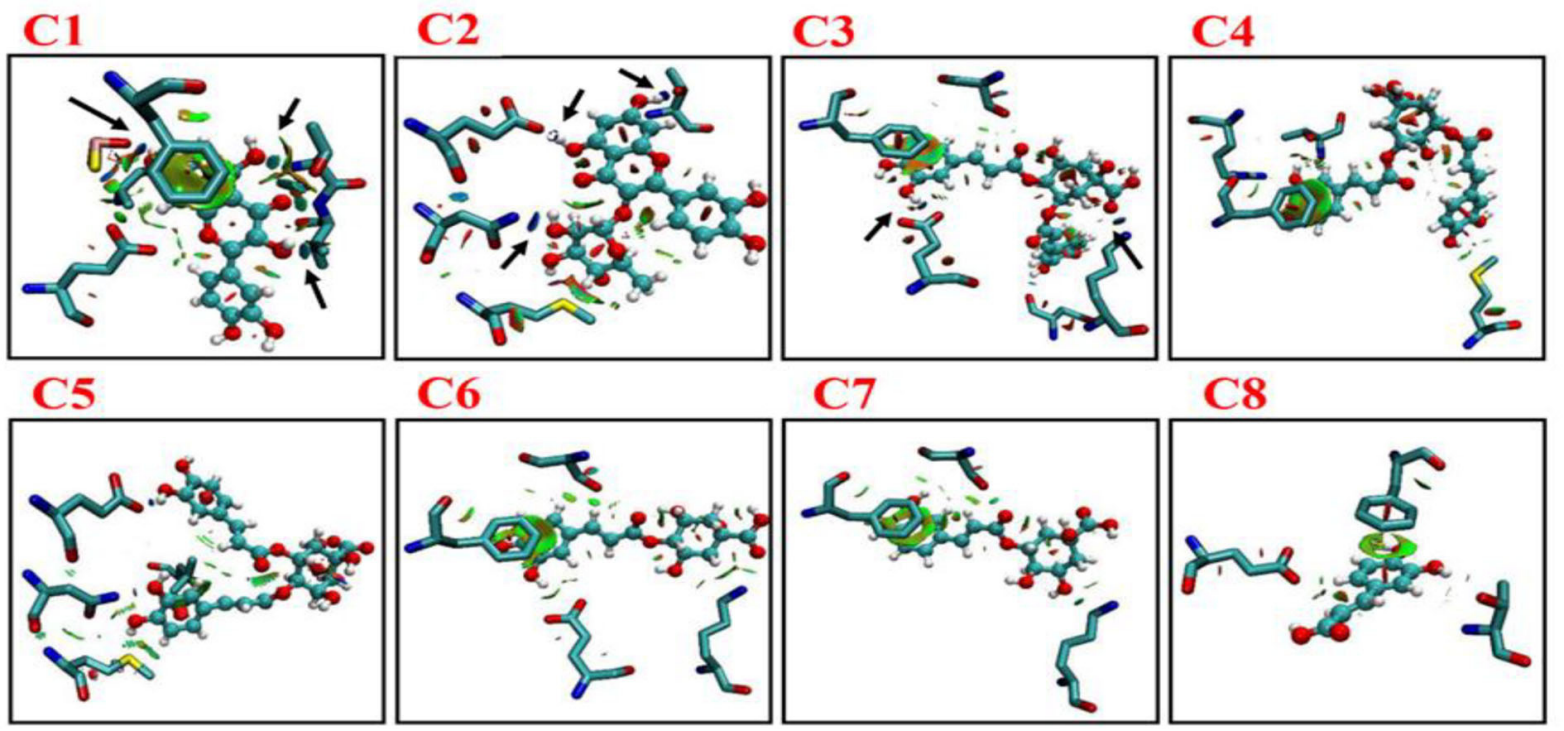
Color mapped RDG isosurface graphs and scatter diagrams of different complexes phenolic compounds (C1**–**C8). Blue represents strong attractive interactions, green represents vdW interactions, and red indicates strong steric effects. C1, quercetin; C2, quercetin-3-rhamnoside; C3, 4,5-O-dicaffeoylquinic acid; C4, 3,5-O-dicaffeoylquinic acid; C5, 3,4-O-di-caffeoylquinic acid; C6, 4-O-caffeoylquinic acid; C7, 3-O-caffeoylquinic acid; and C8, caffeic acid.

The RDG isosurface of the compounds (C1–C8) was plotted by using the molecular modeling software VMD. As presented in Figure 9, evidence showed that strong attraction, steric repulsion, and weaker van der Waal's interaction were formed with surrounding residues. For the C1–C3 system, the RDG analysis revealed the existence of van der Walls (green area) interaction and hydrogen bonds (blue area) with adjacent residues. A hydrogen bond is widely formed in C1, C2, and C3 systems, which were not observed in the C4–C8 systems. For the C4–C8 systems, RDG analysis revealed that π-stacking interactions (green area in isosurfaces) played a major role in the interaction of compounds with surrounding residues.

### Electrostatic potential

**Figure 10:**
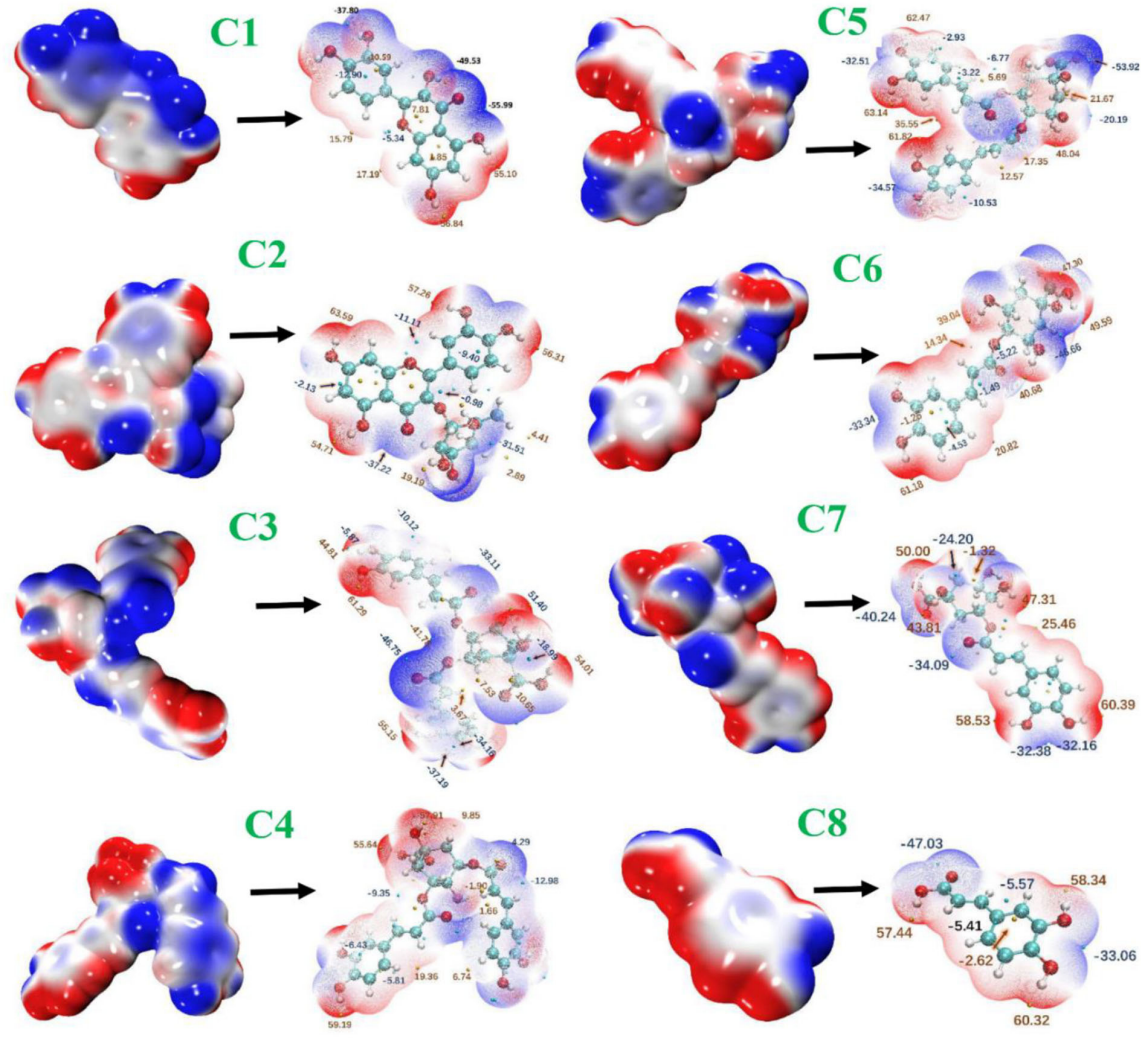
Electrostatic potential surfaces mapped molecular vdW surface of phenolic compounds. The transparent ones correspond to the extrema at the backside of a graph. C1, quercetin; C2, quercetin-3-rhamnoside; C3, 4,5-O-dicaffeoylquinic acid; C4, 3,5-O-dicaffeoylquinic acid; C5, 3,4-O-di-caffeoylquinic acid; C6, 4-O-caffeoylquinic acid; C7, 3-O-caffeoylquinic acid; and C8, caffeic acid.

The ESPs were determined from the electron probability density distribution ρ (*r*) = γ (r, r) and nuclear positions. The ESPs give the idea of binding affinities. It is very helpful for a deeper understanding of key interactions among the compounds (C1–C8) and nearby residues. Electrophiles possess positive charges with empty orbitals which further facilitate attraction to a negative ESP. As a result, minima ESP on the vdW surface tends to be the most important place for electrophilic attack (Figure 10). In the compound (C1), the lone pair of every O_2_ atom results in one or more minima ESP at vdW surface with the most negative one −55.99 kcal/mol (global surface minimum). In compound C1, every surface maximum due to hydrogen, and global surface minimum global maximum one by the positive charge of H3. The distribution of the maximum and minimum values of the electrostatic potential was similar in the remaining seven phenolic compounds.

### Atom in molecules analysis

**Figure 11:**
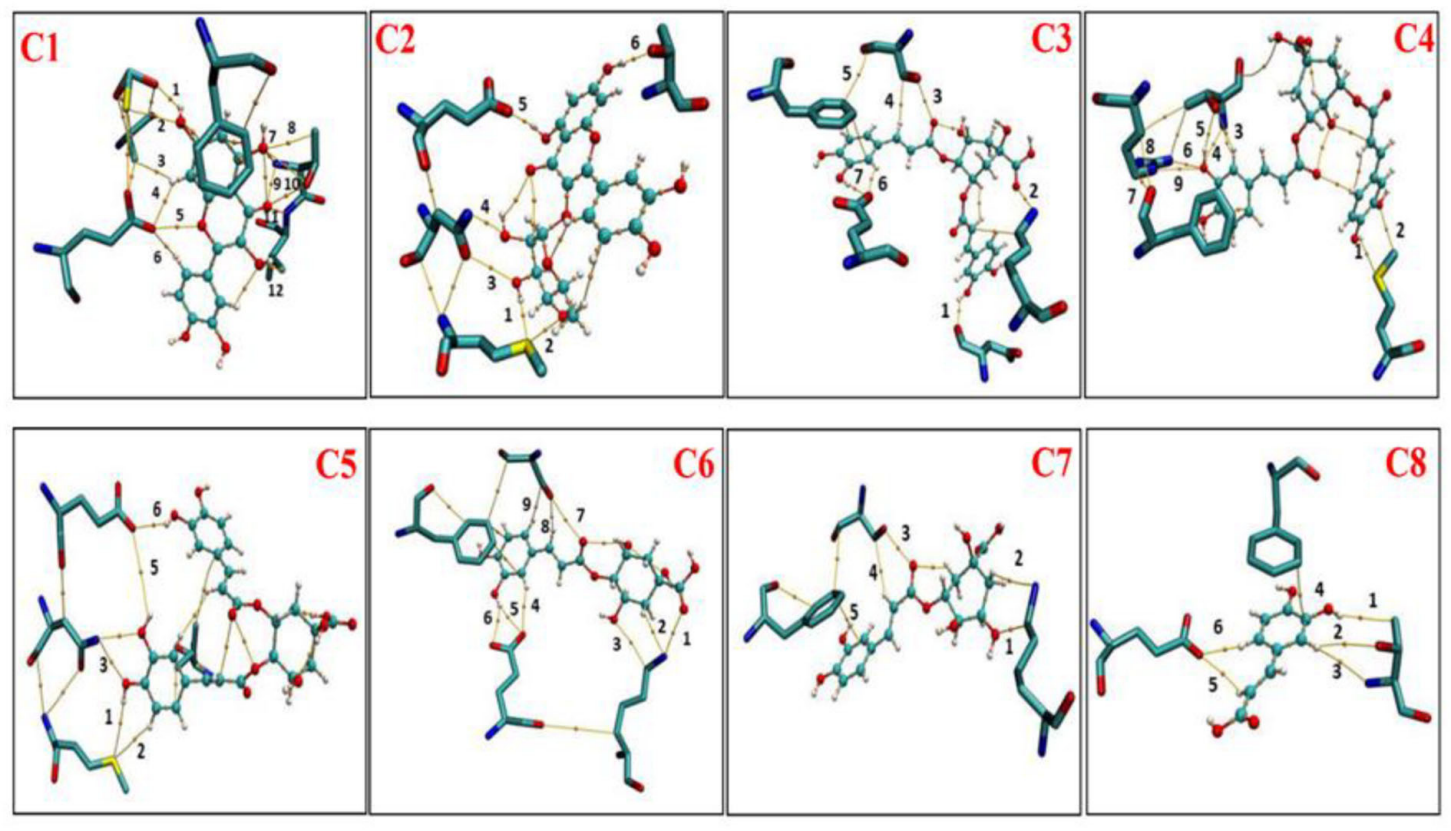
Atom in molecules (AIM) analysis of phenolic compounds (C1-C8) with active residues of XO (Xanthine oxidase). C1, quercetin; C2, quercetin-3-rhamnoside; C3, 4,5-O-dicaffeoylquinic acid; C4, 3,5-O-dicaffeoylquinic acid; C5, 3,4-O-di-caffeoylquinic acid; C6, 4-O-caffeoylquinic acid; C7, 3-O-caffeoylquinic acid; and C8, caffeic acid.

The AIM analysis was carried out at the M06-2x/6-311+G (d, p) level of theory to further clarify the van del Walls and hydrogen bonding interactions. Figure 11 represents that there were topological properties at the bond critical points (BCPs), including the parameters of kinetic energy density (G), electron density (ρ), potential energy density (V), and Laplacian of electron density (∇^2^ρ) at the representative BCPs. Espinosa et al. (19) documented that the BE can be estimated as the half value of V(r) at the BCP for HBs, namely BE ≈ V_BCP_/2. This equation is very famous and commonly employed for the identification of HB strengths. For the criterion of hydrogen bonding, the method proposed by Lipkowski et al. (20) was widely used, which summarized the value of ρ(r_BCP_) 0.02–0.04 a.u. and ∇^2^ρ(r_BCP_) value between 0.02 and 0.15 a.u. As presented in Figure 11, the electron density for C1 at BCP sites 1, 9, 10, and 12 is according to the range necessary for hydrogen-bonding interactions with electron densities ρ(r) value 0.03050, 0.03485, and 0.03823, respectively, and with Laplacian of electron density value 0.1179, 0.1319, 0.1323, and 0.07312, respectively. In C2, ND2_N768_, OE1_E802_, and OG1_T1010_ formed hydrogen bond with C2 with the ρ value 0.03485, 0.02017, and 0.02178, respectively, and ∇^2^ρ values 0.1387, 0.07450, and 0.08475, respectively. In C3, OE1_E802_, and NZ_K771_ formed hydrogen bonds with C3, which corresponding to the BCP sites 2 and 7, with ρ values 0.02815 and 0.02873, respectively, and ∇^2^ρ values 0.09744 and 0.08603, respectively. Compared with C2 and C3 systems, four hydrogen bonds exist in the C1 system, with relatively stronger hydrogen bonding strength, suggesting a stronger interaction between C1 and surrounding residues. When it comes to the C4–C8 system, ρ/∇^2^ρ values are out of the range for hydrogen bond interaction. However, plenty of BCPs was observed in C4 and C6 systems, the values of ρ and ∇^2^ρ were quite minimal, belonging to the weak van der Walls interaction.

## Discussion

The prevalence rate of hyperuricemia (HUA) has significantly increased over the past decades. The people with higher UA levels directly affected renal UA excretion which in turn causes renal damage. Natural products contain a higher amount of polyphenolic compounds, which have been proven for their beneficial effect on human health. In this study, we reported the antihyperuricemic mechanism of eight phenolic compounds, which are commonly present in various food plants through *in-vitro*, and *in-silico* approaches.

XO catalyzes the metabolism of hypoxanthine and xanthine to UA, and XO activation may cause HUA (21). Consequently, inhibition of key enzyme XO is a common method to combat HUA, which can be achieved by allopurinol and febuxostat. However, the main drawback of already-present drugs are potent XO inhibitors (allopurinol and febuxostat) and causes various disorders, such as gastrointestinal problems, skin rashes, fever, hypersensitivity reactions, and worsening of renal function (9, 10). The food plant drives bioactive compounds generally recognize as safe and possesses the ideal structure required for XO inhibition, such as a flexible backbone, hydrophobic nature, and H-bond acceptors and donors (22–27). Notably, there are a lot of variations among the present and previously reported studies that exist against XO with the same compounds. This might be due to the availability of multiple methods for the same enzyme, experimental conditions, and instrument (15, 24–26, 28). For example, Song et al. (29) observed that caffeic acid weakly inhibited (5.74 ± 2.64%) XO at 125 μM concentration, whereas Wang et al. (30) documented that caffeic acid moderately inhibited the XO with IC_50_ of 65.58 ± 2.71 μM. Earlier, Chan et al. (31) reported that the IC_50_ value of caffeic acid was higher than 50 μM against XO. In another study, Chang et al. (32) reported that caffeic acid potently inhibited XO with an IC_50_ of 65.58 μM. Nguyen et al. (33) also observed that caffeic acid strongly inhibited XO with IC_50_ values of 85.3 μM. Recently, Wang et al. (28) observed that caffeic acid abrogated uric acid production via strongly inhibiting XO (IC_50_: 53.45 μM) compared to standard drug allopurinol (IC_50_: 6.96 μM). Hence, it can be concluded that XO inhibitory activity of chlorogenic acid compounds does not rely on the quinic or caffeoyl group. Their conjunction side might play an important role in affecting XO activity. Notably, three di chlorogenic acids (C3, C4, and C5) showed more profound XO inhibitory activity concerning their mono chlorogenic acids (C6 and C7) and caffeic acid (C8).

Similarly, in the case of flavonoids (C1: quercetin and C2: quercetin-3-O-rhamnoside) both possessed strong XO inhibitory activity. However, quercetin (C1) exhibited more potent XO inhibitory activity compared with quercetin-3-O-rhamnoside (C2). This might be due to the additional benzene ring in the structure of quercetin-3-O-rhamnoside (C2) compound, which decreased the effect on XO inhibition. Moreover, our molecular docking study results revealed that both C1 and C2 bound at the molybdenum (MO) active site of XO. However, the results of this study are correlated with the previous studies that flavonoids exhibited higher XO inhibitory activities compared with other classes of polyphenolic compounds (23, 26).

The plant-based foods diet reported to directly link with the lowering incidence of various diseases, including HUA and gout. These biological activities are associated with the presence of bioactive compounds, such as polyphenols. Polyphenols are the most common plant-based functional bioactive components that hold numerous health benefits and utilize for the formulation of nutraceutical and functional foods. Polyphenols from numerous food plants are reported to have the potential to combat hyperuricemic disorder by reducing UA synthesis via XO blocking (16). In this study, we try to explore the protective effect of eight polyphenolic compounds that are commonly present in various food plants. The results of AFM, ^1^H NMR, and computational methods (molecular docking, MD simulation, RGA, SASA, Hirshfeld surface analysis, RDG, electrostatic potential, AIM, and IGM) revealed that polyphenolic compounds (C1–C8) have the potential to combat HUA via blocking the XO enzyme (Figures 1–11 and Supplementary Table 1). However, further preclinical and clinical studies are needed to verify our current study finding and developing a food product that will have benefits for uric acid patients.

## Material and instruments

### Chemicals

Xanthine oxidase (XO), allopurinol, and dimethyl sulfoxide*-*d6 (DMSO-d6) were purchased from Sigma-Aldrich (Shanghai, P. R. China). Quercetin (C1), quercetin-3-rhamnoside (C2), 4,5-O-dicaffeoylquinic acid (C3), 3,5-O-dicaffeoylquinic acid (C4), 3,4-O-di-caffeoylquinic acid (C5), 4-O-caffeoylquinic acid (C6), 3-O-caffeoylquinic acid (C7), and caffeic acid (C8) were purchased from Chengdu Must Biotechnology Co., Ltd. (Chengdu, Sichuan, China). All other chemicals used in this study were analytical grades.

### XO inhibitory activity of phenolic compounds

The XO inhibitory activity of phenolic compounds (C1–C8) was determined according to the previously reported method of Lin et al. (34). In detail, phenolic compounds with various concentrations (50 μl each) were thoroughly mixed with XO enzyme (0.1 U/ml) and incubated at 37°C for 15 min. The reaction was started by the addition of xanthine or hypoxanthine (150 μl). The absorbance was recorded at 292 nm for every 10 s from (0–10 min) by using spectra Max i3 (Molecular Devices). Tris-HCl buffer was used as a negative control, whereas allopurinol was used as a positive control, and XO inhibitory activity was calculated by using the following formula:


(1) $$\begin{array}{l} \text{XO inhibition }\left(\%\right)=\frac{D1-D}{D1}\times 100\ldots \ldots \ldots \ldots \ldots \ldots \end{array}$$

D1 refers to the absorbance of blank, whereas D is the absorbance of tested phenolic compounds (C1–C8). The IC_50_ value at 50% inhibition was calculated by using the linear regression equation in graph pad prism.

### Determination of inhibition type

To determine the mode of inhibition of phenolic compounds toward XO enzyme, a Line-weaver Burk plot analysis was constructed. The XO inhibitory activity was performed as reported above. The kinetic study was conducted in the presence and absence of C1–C8 compounds and calculated by the following equation:


(2) $$\begin{array}{l} \frac{\text{1}}{\text{v}}=\frac{{\text{K}}_{\text{m}}}{{\text{V}}_{\max}}\text{(1 +}\frac{\left[\text{I}\right]}{{\text{K}}_{\text{i}}}\text{)}\frac{\text{1}}{\left[\text{S}\right]}\times \frac{\text{1}}{{\text{V}}_{\text{max}}}\text{……………………..} \end{array}$$

*Km* and *Ki* represent inhibition and Michaelis–Menten constant, *v* is the enzyme reaction rate, and *[S]* and *[I]* inhibitors concentration (26).

### Atomic force microscopic analysis of phenolic compounds (C1–C8) with XO

AFM analysis was performed by using previously published method modifications (35, 36). The 100 μl of XO (0.1 U/ml) was mixed with phenolic compounds (C1–C8) and incubated at 37°C for 30 min. After that, the mixture was added to the mica substrate and dried for 12 h at room temperature. AFM analyses were determined in the air by using Bruker's Dimension® Icon™ Atomic Force Microscope (AFM).

### ^1^H NMR titration assay

The interaction between phenolic compounds (C1–C8) and XO was further investigated by using a ^1^H NMR titration assay. All compounds were dissolved in DMSO-d6 (0.5 ml, 10 mM), titrated with various concentration of XO (0.1–0.5 mg/μl) and ^1^H NMR spectra was recorded (37).

### Computational study of phenolic compounds (C1–C8) with XO

#### Structural preparation for molecular docking analysis of phenolic compounds

The starting structural coordinates of XO were retrieved from an online free server protein data bank (PDB ID:1FIQ, www.rcsb.org). The crystal structures were prepared by assigning binding order, removing water, and adding hydrogen atoms by using Discovery Studio v3.5 software. To avoid the steric clashes, formal charges, non-protein atoms, and binding missing atoms were removed.

#### Molecular docking

Nine crystal structures of the XO in complex with structurally diverse inhibitors from phenolic compounds (**C1–C8**) were retrieved from the PubChem database. XO in complex with allopurinol (standard), and XO in complex with another eight different compounds. After that, structures were prepared according to protein preparation Wizard in Schrödinger, and binding sites were arranged (38). The cross-docking calculations were used for the determination of sampling power. The molecular docking was carried out by using the Glide program. The substrate conformation was evaluated by using CAESAR (39) method encoded in Discovery Studio 3.5 (100 ligand conformations were obtained). The best conformation (25) with low energy was further optimized by the PM6 method (40, 41) in Gaussian09 (42). All native and compared models of different compounds were produced in Chimera by using proteins preparation module (43, 44). The molecular docking grid was specified and centered as per the crystallographic structure, considering the residues Glu802, Phe914, Phe1009, Thr1010, and Val1011 as the active site. After that, grid points were fixed as 20 × 20 × 20 with grid spacing (0.375). The root-mean-square deviation (RMSD) among the crystal structure pose and docking pose was calculated. The higher score confirmation was further used for minimization of energy. Afterward, structures were reproduced by using AMBER18 software package (45).

#### All-atom molecular dynamic simulation

All-atom molecular dynamic (MD) simulation analysis was performed in the AMBER18 software package (45). Each system in a rectangular box was applied to solvate by the TIP3P water model. After that, each neutralized system was subjected to two steps of minimization (heating cycle and the temperature gradually being raised to 298 K). Subsequently, 50 ns MD simulation was conducted after equilibration for 50 ps. The Particle Mesh Ewald (PME) and SHAKE algorithm method were applied for long-range electrostatic interactions and fix bonds and angles involving hydrogen atoms (46, 47).

#### The non-covalent interactions analysis of phenolic compounds and nearby residues

In this study, we used various methods for determining the analysis of the non-covalent interaction of phenolic compounds and nearby residues of XO, such as Hirshfeld surface analysis (48) independent gradient model (IGM) (49), molecular electrostatic potentials, reduced density gradient (RDG) (50), and atoms in molecules (AIM) (45) by using Multiwfn 3.6 program (51) and further visualized by the VMD 1.9.3 program (52).

#### Independent gradient model analysis

It depends on the topological characteristics of the electronic charge density, ρ, of the system under study. The IGM descriptor δg^inter^ is given by the difference between the first derivatives of the charge densities for the total system and the fragments:


(3) $$\begin{array}{l} {\delta g\left(r\right)}^{inter}=|\nabla \rho^{IGM,inter}|-|\nabla \rho |\ldots \ldots \ldots \ldots \ldots \ldots \ldots \end{array}$$

δg^inter^ > 0 indicates the presence of weak interactions and the magnitude of the descriptor at a point in space indicates the strength of the interaction.

#### Independent gradient model, hirshfeld surface, and atom in molecules analysis

The noncovalent interaction (NCI) method, which is also known as the reduced density gradient (RDG) method, is a very popular method to reveal weak interlayer interactions, and it was performed according to the method of Li et al. (39). The Hirshfeld molecular surface was generated by the Multiwfn 3.6 program by using the method reported by McKinnon et al. (53) and Bondi (17). Topology analysis is widely used to analyze real space functions, such as electron density in an atom in molecules (AIM) theory (45).

### Statistical analysis

Data were analyzed by using Graph Pad Prism version 8.0 (California corporation). To compare different groups, we employed one-way ANOVA, followed by Tukey's test at *p* < 0.05 significant level by using Graph Pad Prism version 8.0. The results were presented as mean ± *SD*.

## Conclusion

In conclusion, this study results revealed the potent XO inhibitory activity of eight polyphenolic compounds (C1–C8). The results of *in vitro* XO assay, AFM, ^1^H NMR, and computational methods showed that the inhibitory activity of polyphenolic compounds (C1–C8) on XO relies upon their structure. These results suggested that polyphenolic compounds have a strong ability to overcome the burden of UA level via interacting with important enzyme XO and can be used as a functional ingredient for the development of safe nutraceutical/functional products for the possible treatment of HUA.

## Data availability statement

The original contributions presented in the study are included in the article/Supplementary material, further inquiries can be directed to the corresponding authors.

## Author contributions

AM conceived the idea and perform analysis. AM and JL designed the experiments. AR, RK, IL, IK, FAlt, and SA analyzed the data. AM, LeZ, MU, FAlh, MS, SY, KA, and LiZ wrote and review the manuscript. All authors have read and agreed to the published version of the manuscript.

## Funding

This work was supported by the Joint Program of Beijing Natural Science Foundation and Beijing Municipal Education Commission (KZ202010011016), Opening Project of Key Laboratory of Trace Element and Nutrition, National Health Commission of the Peoples Republic of China (No. wlkfz202205), Research Foundation for Youth Scholars of Beijing Technology and Business University, the project of Discipline Construction-Food Science and Engineering (SPKX-202204), and Taif University Researchers Supporting Project (TURSP-2020/222), Taif University, Taif, Saudi Arabia.

## Conflict of interest

The authors declare that the research was conducted in the absence of any commercial or financial relationships that could be construed as a potential conflict of interest.

## Publisher's note

All claims expressed in this article are solely those of the authors and do not necessarily represent those of their affiliated organizations, or those of the publisher, the editors and the reviewers. Any product that may be evaluated in this article, or claim that may be made by its manufacturer, is not guaranteed or endorsed by the publisher.

## Abbreviations

SASA: solvent accessible surface area
RMSD: root-mean-square deviation
FAD: flavin adenine dinucleotide
Mo: molybdenum
AFM: Atomic force microscopic
H and E: hematoxylin and eosin
XO: Xanthine oxidase
XDH: xanthine dehydrogenase
CMC-Na: Sodium carboxymethyl cellulose
AIM: atoms in molecules
RDG: reduced density gradient
IGM: Independent gradient model
MD: Molecular dynamic
UA: uric acid
BUN: blood urea nitrogen
ESPs: Electrostatic potential
BCPs: bond critical points
HUA: hyperuricemia.
