# Supplementary material for: Xanthine oxidase inhibitory study of eight structurally diverse phenolic compounds
Source: Front Nutr. 2022 Sep 20;9:966557. doi: 10.3389/fnut.2022.966557 (PMC9531272; doi:10.3389/fnut.2022.966557)
Supplement: Supplementary file 1 [file Table_1.DOCX]

**Table 1. Comparison of the Values of Topological Descriptors**

| **Compound** | **Type** | **ρ(r) (a.u.)** | **∇^2^ρ(r) (a.u.)** | **G(r) (a.u.)** | **V(r) (a.u.)** |
| --- | --- | --- | --- | --- | --- |
| C1 | 1 | 0.03050 | 0.1179 | 0.02867 | -0.02787 |
|  | 2 | 0.01277 | 0.04464 | 0.01010 | -0.00904 |
|  | 3 | 0.00257 | 0.00916 | 0.00164 | -0.00099 |
|  | 4 | 0.01217 | 0.0401 | 0.00932 | -0.00862 |
|  | 5 | 0.00982 | 0.0350 | 0.00836 | -0.00797 |
|  | 6 | 000644 | 0.02468 | 0.00517 | -0.00417 |
|  | 7 | 0.02284 | 0.07728 | 0.01714 | -0.07728 |
|  | 8 | 0.00868 | 0.03137 | 0.00685 | -0.00586 |
|  | 9 | 0.03485 | 0.1387 | 0.03479 | -0.03489 |
|  | 10 | 0.03823 | 0.1323 | 0.03997 | -0.04686 |
|  | 11 | 0.01916 | 0.07070 | 0.01566 | -0.01364 |
|  | 12 | 0.02642 | 0.07312 | 0.01760 | -0.01691 |
| C2 | 1 | 0.01860 | 0.09672 | 0.02928 | -0.02939 |
|  | 2 | 0.03473 | 0.1320 | 0.03592 | -0.03882 |
|  | 3 | 0.00225 | 0.02541 | 0.00444 | -0.00253 |
|  | 4 | 0.02017 | 0.07450 | 0.01622 | -0.1382 |
|  | 5 | 0.01789 | 0.0819 | 0.02208 | -0.02119 |
|  | 6 | 0.02178 | 0.08475 | 0.01887 | -0.01656 |
| C3 | 1 | 0.01831 | 0.09439 | 0.02889 | -0.02919 |
|  | 2 | 0.02815 | 0.09744 | 0.02585 | -0.02733 |
|  | 3 | 0.00763 | 0.05462 | 0.01098 | -0.00831 |
|  | 4 | 0.00556 | 0.01803 | 0.00353 | -0.00255 |
|  | 5 | 0.00099 | 0.00550 | 0.00092 | -0.00046 |
|  | 6 | 0.00817 | 0.04189 | 0.00784 | -0.00521 |
|  | 7 | 0.02873 | 0.08603 | 0.02537 | -0.02924 |
| C4 | 1 | 0.00848 | 0.03765 | 0.00699 | -0.00457 |
|  | 2 | 0.00863 | 0.03456 | 0.00716 | -0.00568 |
|  | 3 | 0.00847 | 0.04218 | 0.00807 | -0.00560 |
|  | 4 | 0.00456 | 0.02822 | 0.00522 | -0.00339 |
|  | 5 | 0.00702 | 0.04138 | 0.00760 | -0.00486 |
|  | 6 | 0.00136 | 0.01379 | 0.00237 | -0.00129 |
|  | 7 | 0.00158 | 0.01131 | 0.00193 | -0.00104 |
|  | 8 | 0.00203 | 0.01347 | 0.00239 | -0.00142 |
|  | 9 | 0.00094 | 0.01061 | 0.00176 | -0.00088 |
| C5 | 1 | 0.00181 | 0.00554 | 0.00101 | -0.000615 |
|  | 2 | 0.00197 | 0.00713 | 0.00120 | -0.00062 |
|  | 3 | 0.00559 | 0.03221 | 0.00614 | -0.00423 |
|  | 4 | 0.00672 | 0.04034 | 0.00811 | -0.00613 |
|  | 5 | 0.12E-04 | 0.17E-03 | 0.26E-04 | -0.808E-05 |
|  | 6 | 0.00543 | 0.03118 | 0.00526 | -0.00273 |
| C6 | 1 | 0.01279 | 0.04345 | 0.009857 | -0.0088 |
|  | 2 | 0.00878 | 0.03266 | 0.00657 | -0.00497 |
|  | 3 | 0.00253 | 0.01058 | 0.00199 | -0.00134 |
|  | 4 | 0.00499 | 0.0184 | 0.00367 | -0.00272 |
|  | 5 | 0.00665 | 0.02418 | 0.00506 | -0.00407 |
|  | 6 | 0.00671 | 0.02498 | 0.00528 | -0.00431 |
|  | 7 | 0.00399 | 0.01767 | 0.00367 | -0.00293 |
|  | 8 | 0.00957 | 0.03428 | 0.00760 | -0.66395 |
|  | 9 | 0.00278 | 0.00862 | 0.00162 | -0.00107 |
| C7 | 1 | 0.01649 | 0.04854 | 0.01143 | -0.01072 |
|  | 2 | 0.00327 | 0.01074 | 0.00210 | -0.00152 |
|  | 3 | 0.01515 | 0.04930 | 0.0120 | -0.01169 |
|  | 4 | 0.00385 | 0.01324 | 0.00243 | -0.00154 |
|  | 5 | 0.00858 | 0.02477 | 0.00501 | -0.00383 |
| C8 | 1 | 0.00302 | 0.01320 | 0.00231 | -0.00131 |
|  | 2 | 0.00095 | 0.00599 | 0.00092 | -0.00035 |
|  | 3 | 0.00067 | 0.00443 | 0.00073 | -0.00036 |
|  | 4 | 0.00701 | 0.01909 | 0.00446 | -0.00415 |
|  | 5 | 0.00547 | 0.02903 | 0.00540 | -0.00355 |
|  | 6 | 0.01072 | 0.04993 | 0.00995 | -0.00741 |

*Note*: Electron Density (ρ), Laplacian of the Electron Density (∇^2^ρ), Potential Energy Density (V(r)) obtained for the BCPs between compound and nearby residues in C1-C8 system
